# Supplementary material for: COVID-19 Bivalent Booster in Pregnancy: Maternal and Neonatal Antibody Response to Omicron BA.5, BQ.1, BF.7 and XBB.1.5 SARS-CoV-2
Source: Vaccines (Basel). 2023 Aug 28;11(9):1425. doi: 10.3390/vaccines11091425 (PMC10537670; doi:10.3390/vaccines11091425)
Supplement: Supplementary file 1 [file vaccines-11-01425-s001.zip › vaccines-2546626-supplementary.pdf]

Table S1. Neutralizing antibody inhibition in maternal and cord blood for Omicron BA.5, BF.7, BQ.1, and XBB.1.5 SARS-CoV-2.

	**BA.5**	**BF.7**	**BQ.1**	**XBB.1.5**	**p value**
Maternal Blood (%)[a]	90.29	96.46	89.76	90.57	0.130
Cord Blood (%)[b]	84.52	89.07	82.11	83.69	0.946
Ratio	0.94	0.92	0.93	0.93	0.635

[a] 2 cases were not included due to the inability to detect any neutralizing antibody inhibition.

[b] 2 cases were not included due to no detection of neutralizing antibody inhibition; 1 case had no calculated values for cord blood due to the lack of an available sample.
